# The relationship between executive function, neurodevelopmental disorder traits, and academic achievement in university students

**DOI:** 10.3389/fpsyg.2022.958013

**Published:** 2022-09-02

**Authors:** Chloe Southon

**Affiliations:** Department of Psychology, Social Work and Counselling, School of Human Sciences, University of Greenwich, London, United Kingdom

**Keywords:** executive function, autism spectrum disorder, attention-deficit hyperactivity disorder, developmental coordination disorder, academic achievement, ASD, ADHD, DCD

## Abstract

Difficulties with executive function have often been identified in individuals with various neurodevelopmental disorders such as Autism Spectrum Disorder (ASD), Attention-Deficit Hyperactivity Disorder (ADHD), and Developmental Co-ordination Disorder (DCD). Additionally, in childhood and adolescence, executive functioning is an important predictor of academic achievement. However, less research has explored these relationships in adult students, and those with a high level of neurodevelopmental disorder traits but no clinical diagnosis. Therefore, the current study aimed to assess whether ASD, ADHD, and DCD traits can predict academic achievement in university students, and whether traits of these neurodevelopmental conditions moderate the relationship between executive function and academic achievement. Both neurotypical students and those with a clinical diagnosis of a neurodevelopmental disorder were able to participate, with the majority being neurotypical. Participants completed four self-report questionnaires and provided a measure of academic achievement based on their university assignment results. Traits of ASD, ADHD, and DCD alone did not predict achievement, however, traits of ADHD and DCD significantly moderated the relationship between executive function and academic achievement. ASD traits did not significantly moderate this relationship. Implications and suggestions for future research are also discussed.

## Introduction

Executive functions are cognitive abilities that are responsible for co-ordinating problem solving and achieving goals (Jurado and Rosselli, 2007; Best and Miller, 2010; Pascual et al., 2019; Zelazo and Carlson, 2020), and are typically used in new or challenging situations (Huizinga et al., 2018). It is widely considered that there are three core executive functions; inhibition, cognitive flexibility, and working memory (Miyake et al., 2000; Best and Miller, 2010; Diamond, 2013). Inhibition refers to the ability to self-regulate and refrain from acting on impulse (Bull and Scerif, 2001; Jurado and Rosselli, 2007; Diamond, 2013), and working memory involves the concurrent processing, maintenance, and recollection of information (Simone et al., 2018; Carrasco et al., 2021). Cognitive flexibility is defined as being able to adapt to different tasks and think creatively (Bull and Scerif, 2001; Diamond, 2013).

### Executive function and academic achievement

There is a consensus among the literature that better executive functioning is associated with higher academic achievement in childhood and adolescence (Bailey et al., 2018). Working memory specifically has been found to predict mathematic ability (Espy et al., 2004; Simone et al., 2018; Carrasco et al., 2021). Further to this, reading and writing skills are also associated with inhibitory control (St Clair-Thompson and Gathercole, 2006; Monette et al., 2011), cognitive flexibility (Bull and Scerif, 2001), and working memory (Blair and Razza, 2007; O’Toole et al., 2018). In addition to this, Clark et al. (2010) found that executive function was a better predictor of academic achievement than IQ in school-age children. Poorer executive functioning is often found in children with poorer academic ability and learning difficulties (Sikora et al., 2002; Thorell et al., 2013). Wray et al. (2020) found that executive functioning is also associated with schooling in adults in low- and middle-income countries using data collected from three different continents, suggesting that the link between executive function and academic achievement is present across different cultures, countries, and socio-economic backgrounds.

Furthermore, previous research has found evidence that executive function may be impaired in individuals with neurodevelopmental disorders (Otterman et al., 2019), including autism spectrum disorder (ASD; Christ et al., 2011; Adams and Jarrold, 2012; Murphy et al., 2014), attention-deficit hyperactivity disorder (ADHD; Arnsten and Li, 2005; Wilens and Spencer, 2010), and developmental coordination disorder (DCD; Leonard et al., 2015; Bernardi et al., 2017). Since difficulties with executive functioning predict poorer academic achievement (Zelazo and Carlson, 2020), and executive function impairments seem to occur in neurodevelopmental disorders such as autism, ADHD, and DCD (Holst and Thorell, 2019; Omer and Leonard, 2021), individuals with these conditions may be particularly at risk of academic underachievement.

If executive functioning can predict reading, writing, and mathematics ability (St Clair-Thompson and Gathercole, 2006; Christopher et al., 2012), and interventions that improve executive functioning are not provided for those with difficulties or such difficulties are not identified at all, these problems may be carried forward long-term. Since executive function can predict long-term academic performance (Samuels et al., 2016), this may result in individuals not reaching their full academic potential. This is of high importance as both executive function and academic achievement can have a direct influence on many areas of life later on such as employment (Bailey, 2007), wellbeing (Bucker et al., 2018), life satisfaction (Brown and Landgraf, 2010; Clark and Malecki, 2019), mental health (Wray et al., 2020), and academic self-esteem (Giofre et al., 2017).

### Executive function and academic achievement in autism spectrum disorder

Autism spectrum disorder is a neurodevelopmental disorder which is associated with impairments in social interaction and communication (Barendse et al., 2013; Milgramm et al., 2021). Individuals with ASD may be at a higher risk of difficulties with academic performance compared to those who are neurotypical (St John et al., 2018; Milgramm et al., 2021). Evidence suggests this may not necessarily be due to intellectual difficulty (Charman et al., 2011), but rather as a result of difficulty managing repetitive behaviors and poor social skills, which may affect their academic performance (Milgramm et al., 2021). Research has often associated academic achievement with IQ (Watson and Monroe, 1990; Mayes and Calhoun, 2003; Mayes et al., 2009). However, Estes et al. (2011) highlighted that there are inconsistencies in IQ and academic performance in autistic children which are not seen in typically developing children, suggesting that there are factors affecting academic achievement in autistic individuals other than IQ, such as executive function (St John et al., 2018).

Working memory has been found to consistently predict academic achievement in autistic individuals (Miller et al., 2017; Kim et al., 2018), with executive function as a whole recently emerging as a potential predictor of academic performance in those with ASD (St John et al., 2018). The link between autism and executive function impairments is well-established (Demetriou et al., 2019; Vaidya et al., 2019; Zhang et al., 2020), however, a recent paper by Lee et al. (2021) found that executive function was not associated with ASD trait severity, but was instead significantly associated with the degree of severity of ADHD traits, a neurodevelopmental disorder that is often co-occurring with ASD (Antshel et al., 2016; Rau et al., 2020; Zablotsky et al., 2020), and is also associated with poorer academic achievement (Holst and Thorell, 2019; Carrasco et al., 2021), similarly to ASD.

### Executive function and academic achievement in attention-deficit hyperactivity disorder

Attention-deficit hyperactivity disorder is characterized by hyperactivity, impulsive behaviors, and being easily distracted (Clark et al., 2002; Simone et al., 2018), and children with ADHD tend to be further behind with mathematics, reading, and writing compared with their typically developing peers (Lawrence et al., 2020). There is some research suggesting that while the severity of ADHD traits may improve over time (Murray et al., 2017), difficulties relating to academic performance may continue and even become worse with age in individuals with ADHD (Barbaresi et al., 2007). This suggests that there may be another factor in the relationship between ADHD and academic achievement, such as executive function impairments (Arnsten and Li, 2005; Martel et al., 2007; Wilens and Spencer, 2010).

There is evidence that academic performance in individuals with ADHD can be predicted by executive functions; verbal working memory ability can predict academic achievement consistently across various academic subjects in those with ADHD (Carrasco et al., 2021) and visual-spatial working memory can predict variance in mathematics ability (Simone et al., 2018; Carrasco et al., 2021). Biederman et al. (2004) observed that children with an ADHD diagnosis were significantly more likely to have executive function impairments than typically developing children, and that children with ADHD and executive function difficulties were more at risk of poorer academic achievement.

These relationships can be seen in adults as well as children; executive function impairments are also associated with academic achievement in university students with ADHD (Gropper and Tannock, 2009), although there is substantially less research in this area with adult participants. Difficulties with executive function can affect individuals with ADHD in many areas of life; such as having poorer theory of mind ability (Tatar and Cansiz, 2020), higher stress levels (McLuckie et al., 2021), and poorer sleep quality (Holingue et al., 2021). If executive function deficits in ADHD are associated with academic performance, then individuals with ADHD may have a higher risk of academic underachievement (Arnold et al., 2020; Carrasco et al., 2021) compared with their typically developing peers, and understanding this relationship may be crucial in determining the contribution of executive function to academic achievement when developing effective interventions for individuals with ADHD, as there is evidence to suggest that better executive function ability can predict adaptive behaviors in those with ADHD (Clark et al., 2002).

### Executive function and academic achievement in developmental co-ordination disorder

Autism spectrum disorder and attention-deficit hyperactivity disorder can also co-occur with Developmental Co-ordination Disorder (DCD; Tal-Saban and Kirby, 2019; Miller et al., 2021). DCD is a neurodevelopmental condition involving difficulties with motor co-ordination (Zwicker et al., 2012; Omer and Leonard, 2021), and is thought to affect 5–6% of children (Zwicker et al., 2012; American Psychiatric Association, 2013). Individuals with autism and ADHD can perform poorly on tasks that assess motor skills even if they do not meet the diagnostic criteria for DCD (Lindor et al., 2019; Miller et al., 2021). These motor impairments can increase with age (Licari et al., 2020), and research by Tal-Saban et al. (2014) found that three-quarters of young adults who had DCD also met diagnostic criteria for ADHD.

Furthermore, motor difficulties in children just 6 months old may predict an ASD diagnosis later on (LeBarton and Landa, 2019), and Miller et al. (2021) found that 90% of the participants with an ASD diagnosis in their study met the criteria for co-occurring dyspraxia, a term often used interchangeably with DCD in the United Kingdom. Children with an ADHD diagnosis who also have poor motor skills may show higher levels of autistic traits (Reiersen et al., 2008), suggesting that there may be a degree of overlap in traits between these conditions. Despite this, Tal-Saban and Kirby (2019) argue that DCD is not as well-understood compared with ASD and ADHD. The participants in the study by Reiersen et al. (2008) were assessed based on the criteria in the DSM-IV used at the time; they suggested a change to these criteria to acknowledge the overlapping symptoms between ASD, ADHD, and DCD, and the DSM-5 now allows for co-occurring ASD and DCD to be diagnosed (Tal-Saban and Kirby, 2019).

Similarly to ASD and ADHD, DCD has often been associated with executive function impairments (Bernardi et al., 2017; Sartori et al., 2020; Omer and Leonard, 2021) and poorer academic achievement (Harrowell et al., 2018; Fastame, 2020; Smits-Engelsman et al., 2021). Executive functions are considered to be regulated by the pre-frontal cortex (Ball et al., 2011; Rosen et al., 2019), a part of the brain involved in higher-order cognitive processes that has extensive connections to other brain areas such as the cerebellum (Diamond, 2000; Leonard and Hill, 2015), which is involved in coordinating movement (Tal-Saban et al., 2014; Leonard and Hill, 2015). These neural connections support the evidence that executive function could be impaired in those with DCD.

Furthermore, motor skills can contribute to academic achievement in both the typically developing population (Davies et al., 2016; Fastame, 2020) and individuals with DCD (Cadoret et al., 2018; Macdonald et al., 2020), therefore impairments in motor skills such as those seen in DCD could potentially be associated with poorer academic achievement (Lopes et al., 2013). Motor impairments appear to be associated with poorer reading and mathematics ability (Macdonald et al., 2020), and children with DCD seem to have poorer mathematics and language ability compared with typically developing individuals (Lopes et al., 2013).

The motor difficulties seen in DCD have been associated with executive function impairments (Leonard et al., 2015; Sartori et al., 2020); children with DCD perform worse on inhibition tasks compared with typically developing children (Pratt et al., 2014). If motor impairments such as those seen in DCD are associated with the development of executive functioning (Fastame, 2020), and if poorer executive function ability is associated with poorer academic achievement (Willoughby et al., 2019; Zelazo and Carlson, 2020), individuals with DCD or with higher levels of DCD traits may be at risk of academic underachievement.

### Findings in adults and those without a clinical diagnosis

Despite numerous papers highlighting the contribution of executive function to academic achievement in those with neurodevelopmental disorders, less is known about this area in adults, as the vast majority of this research has been conducted with children. Some research has begun to address the contribution of executive function to academic achievement in adults with neurodevelopmental disorders; DCD and dyslexia have been related to lower academic confidence in university students with these conditions (Sumner et al., 2021), and there is evidence that working memory deficits can impair academic performance in university students with ADHD (Gropper and Tannock, 2009). Additionally, co-occurring ADHD and DCD is associated with lower educational levels in adults (Rasmussen and Gillberg, 2000). Executive function may also have a crucial role in academic achievement in university students with ASD as both autistic traits and executive functioning can significantly predict academic progress (Dijkhuis et al., 2020). However, there appears to be a lack of literature on individuals with high levels of traits of neurodevelopmental disorders, rather than a clinical diagnosis.

It may be possible for individuals to have high levels of traits of a neurodevelopmental condition despite lacking a clinical diagnosis (Constantino and Todd, 2003; Loe and Feldman, 2007). For example, children who demonstrate difficulties with hyperactivity and inattention but no clinical diagnosis of ADHD can have poor academic achievement (Loe and Feldman, 2007), and Fastame (2020) concluded that motor skills are critical to academic achievement in both neurotypical individuals as well as those with DCD. Fastame (2020) suggests that the underlying mechanism for this develops early on in infancy; playing and interacting with physical objects develops sensory-motor intelligence, which Fastame (2020) suggests is of utmost importance for further cognitive development. Based on this research, it seems likely that high levels of neurodevelopmental disorder traits that do not reach the threshold for a clinical diagnosis could affect academic achievement in university students, and with the literature being consistent that executive function impairments are associated with neurodevelopmental disorders, it may be possible that levels of neurodevelopmental disorder traits have a role in the relationship between executive function and academic achievement.

If neurodevelopmental disorder traits are found to have a significant role in the relationship between executive function and academic achievement in university students, this could result in potential changes to intervention strategies for individuals with these conditions or those with high levels of neurodevelopmental disorder traits. It could also perhaps improve access to better quality support for students with such conditions or traits, especially for those with a high level of ASD, ADHD, or DCD traits who may otherwise be missed due to the lack of a diagnosis. Additionally, identifying high levels of neurodevelopmental disorder traits in students may provide prognostic value; it may indicate that a student could potentially be at risk of academic underachievement.

Thus, if these traits are identified early, any necessary interventions such as those that seek to improve executive functioning can be put in place for the student earlier on, giving them a higher chance of academic success (for a review, see Jacob and Parkinson, 2015). Such findings could also enhance current understanding of how high levels of neurodevelopmental disorder traits in adult students with no clinical diagnosis relates to their academic achievement, which is essential for long-term life outcomes (Giofre et al., 2017), such as self-esteem (Alves-Martins et al., 2002), life satisfaction (Clark and Malecki, 2019), and wellbeing (Bucker et al., 2018).

### The present study

The links between executive function and academic achievement, and between neurodevelopmental disorders and academic achievement, are well-established in the literature. However, less is known about these relationships in adult university students and in those with a high level of neurodevelopmental traits but no clinical diagnosis. In addition, there still remains a gap in the literature regarding the nature of this relationship, for example, the role of neurodevelopmental disorder traits in the relationship between executive function and academic achievement. Therefore, the current study seeks to assess whether traits of ASD, ADHD, and DCD predict academic achievement in university students as the first aim. Then, considering the potential EF deficit associated with neurodevelopmental disorders, the study will additionally consider the contribution of EF by using moderation analysis to assess whether traits of ASD, ADHD, and DCD moderate the relationship between executive function and academic achievement as the second aim. The hypotheses are;

1. Higher levels of ASD, ADHD, and DCD traits will significantly predict lower academic achievement in university students.
2. Higher levels of ASD, ADHD, and DCD traits will significantly moderate a positive relationship between executive function and academic achievement in university students.

## Method

### Participants

One hundred and seventy-five university students took part in this study, however, there were only one hundred and fifty-eight completed responses that were used for the analysis. Of these 158 participants, 87.3% were female (*N* = 138), 10.8% were male (*N* = 17). 1.9% of participants preferred not to state their gender (*N* = 3). The age range was 18–47 (*M* = 21.31, *SD* = 4.07). Twenty-one participants had a diagnosis of at least one neurodevelopmental condition; nine had a diagnosis of autism, nine had ADHD, two had attention-deficit disorder (ADD), seven had dyslexia, and one had dyspraxia. Of these, six participants had co-occurring neurodevelopmental disorders; three had co-occurring ASD and ADHD, one had ASD and ADD, one had ADHD and dyslexia, and one had ADHD, DCD, and dyslexia. The remaining participants (*N* = 137) were neurotypical, therefore, this was a majority non-clinical sample. To be eligible to participate in this study, participants had to be university students aged 18 and above. This included both undergraduates and postgraduates, full time or part time. However, they had to be on taught programs to be able to participate, therefore, PhD students were not included.

Participants did not need to have a diagnosis of any neurodevelopmental disorders to take part; both neurotypical and neurodiverse students were eligible for participation. However, they were asked whether they have a diagnosed neurodevelopmental condition and to specify which, to be sure that any significant results found were not due to a large proportion of participants having clinical diagnoses of the neurodevelopmental disorders of interest. Neurodiverse participants were included as well as neurotypical individuals for two reasons; the first was to ensure variation in scores, since individuals with a clinical diagnosis of a neurodevelopmental disorder are likely to have higher levels of traits according to the diagnostic criteria of the DSM-5 (American Psychiatric Association, 2013).

The second reason was to ensure the sample would reflect the true spread of neurotypical and neurodiverse individuals in a sample of university students. This was important to consider given that increasing numbers of individuals with neurodevelopmental conditions, such as ASD, are opting to progress to postsecondary education (Dijkhuis et al., 2020). Participants were recruited using the online platforms SONA, which recruited students from the researcher’s institution and where the majority of participants were recruited from, and Call for Participants, which opened up the study to university students from institutions across the globe. Other university students known personally to the researcher were also invited to participate. Participants were not required to disclose which course they were studying or at which university. Participants who may be likely to experience distress as a result of the topics included in the study were advised not to participate.

### Design and statistical analyses

This study used a correlational design, and was conducted online using the Qualtrics platform. Data were first checked for normality using histograms, and all variables followed a normal distribution shape. However, ASD and DCD traits displayed a very slight positive skew. This means that on average, participants generally had slightly lower levels of ASD and DCD traits. A Pearson correlation matrix was used to assess correlations between all variables. A multiple regression was used to determine whether levels of ASD, ADHD, and DCD traits predict academic achievement, and a moderation analysis using the PROCESS macro model 1 was used to assess whether ASD, ADHD, and DCD traits, respectively, moderated the relationship between executive function and academic achievement. All analyses were completed in IBM SPSS Statistics Version 27.

### Ethical considerations

This study was granted ethical approval from the ethics committee at University of Greenwich. Participants were asked to give informed consent before beginning the study, which detailed what they would be asked to do, and assured them that participation is voluntary. Participants could withdraw from the study at any time, and they could withdraw their data for up to a week after they completed the study. They were given the researcher’s email address if they had any questions or concerns, or if they wished to withdraw their data, by stating their unique personal code they created at the start to ensure confidentiality.

There was no risk of physical harm to participants, however, there may have been a very small risk of psychological distress. This may have occurred as a result of the nature of some of the questionnaire items; disclosing the results of their university assessments may raise concerns relating to self-esteem and others may be sensitive toward research into neurodevelopmental disorders. To reduce the likelihood of psychological distress, participants were advised not to take part if they are sensitive to the topics being studied, and they were given the details of the university counseling service and other organizations relating to autism, ADHD, and DCD on the debrief form.

### Materials

This was an online study which required the use of an internet-capable device and an internet connection. Data were collected across two academic years; 2020–2021 and 2021–2022. Self-report data was obtained from four questionnaires. Executive functioning was determined by scores on the Amsterdam Executive Function Inventory (AEFI; Van der Elst et al., 2012), consisting of 13 items assessing executive function using a three-point scale consisting of *not true* (1), *partly true* (2), or *true* (3). This scale was chosen over laboratory methods due to the convenience of it being able to be completed by a large sample, and it may also provide a better insight into executive functions in everyday life rather than laboratory-based methods which may lack ecological validity somewhat (Leonard and Hill, 2015).

ASD traits were measured using the Autism Quotient (AQ; Baron-Cohen et al., 2001), which consists of 50 items regarding autism-typical behaviors on a four-point scale consisting of *definitely agree* (1), *slightly agree* (2), *slightly disagree* (3), or *definitely disagree* (4). *Definitely agree* and *slightly agree* responses were scored 1 point on autism-typical items such as “I prefer to do things the same way over and over again.” There were 26 reverse scored items, such as “I prefer to do things with others rather than on my own,” where *slightly disagree* and *definitely disagree* were scored 1 point.

ADHD traits were measured using the Adult ADHD Self-Report Scale (ASRS; Kessler et al., 2005), which has 18 items relating to ADHD traits. Participants indicated how often each item occurs in their life on a five-point scale from *never* (1) to *very often* (5). DCD traits were measured using the Adult DCD Checklist (ADC; Kirby et al., 2010), which has 30 items relating to motor coordination. This measure is divided into two sections; childhood (10 items) and present time (20 items). Participants indicated how often these items apply to them, on a four-point scale from *never* (1) to *always* (4). Responses on all measures were scored summed to provide a total score.

Academic achievement was measured using students’ results from the first assignment and most recent assignment they submitted during the academic year in which they participated. These scores were used to calculate a mean to provide a measure of academic achievement. In the event that a participant had only completed one assignment during the academic year at the time of participating, they were asked to provide the same number as they did for the previous question. Items across all measures were scored such that higher scores meant better executive functioning, higher levels of ASD, ADHD, and DCD traits, and higher academic achievement.

### Procedure

Participants were asked for demographic data (age and gender), and whether or not they have a neurodevelopmental disorder. If so, they were asked to specify which. Participants were then asked for the result of their first university assessment and most recent assessment of the academic year in numerical form out of 100, as a measure for academic achievement. Participants then completed the AQ (Baron-Cohen et al., 2001), ASRS (Kessler et al., 2005), the ACD (Kirby et al., 2010), and the AEFI (Van der Elst et al., 2012), and were then fully debriefed.

## Results

The means, standard deviations, and range of scores for each measure are shown in Table 1. Correlations between all variables are presented in Table 2.

**TABLE 1:** Means, standard deviations, and range of scores across each measure.

Measure	*M*	*SD*	Minimum	Maximum
Autism quotient (AQ)	20.85	8.08	7	50
ADHD self-report scale	52.35	12.65	23	89
DCD checklist	57.27	15.64	30	109
Amsterdam executive function inventory (AEFI)	27.28	4.28	16	37
Academic achievement	62.44	8.27	42	100

N = 158.

**TABLE 2:** Correlation matrix of variables.

Measure	2.	3.	4.	5.
1. Autism quotient	0.475**	0.472**	–0.212**	–0.94
2. ADHD self-report scale		0.632**	–0.163*	0.047
3. DCD checklist			–2.17**	–0.015
4. Amsterdam executive function inventory				–0.128
5. Academic achievement				

N = 158. *p < 0.05, **p < 0.01.

A multiple regression revealed that ASD, ADHD, and DCD traits accounted for 2% of variance in academic achievement, *R*^2^ = 0.02, *F*(3, 154) = 1.06, *p* = 0.367, but this was non-significant. Individually, traits of ASD β = –0.14, *t*(154) = –1.52, *p* = 0.13; ADHD β = 0.14, *t*(154) = –1.27, *p* = 0.205, and DCD β = –0.03, *t*(154) = –3.12, *p* = 0.75, did not significantly predict academic achievement.

Three moderated regression analyses were performed using the PROCESS macro model 1, with executive function as the predictor, academic achievement as the outcome, and traits of ASD, ADHD, and DCD as the moderator in each regression, respectively. All variables were centered, and the conditioning values were set to –1 SD, mean, +1 SD. With ASD traits as the moderator, 5% of the variance in academic achievement was explained, *R*^2^ = 0.05, *F*(3, 154) = 2.82, *p* = 0.04. However, the interaction was non-significant [β = 0.04, *t*(154) = 1.82, *p* = 0.07].

With ADHD traits as the moderator, 5% of the variance in academic achievement was explained, *R*^2^ = 0.05, *F*(3, 154) = 2.94, *p* = 0.03. The interaction between executive function and ADHD traits was significant [β = 0.02, *t*(154) = 2.46, *p* = 0.01], suggesting that ADHD traits significantly moderated the relationship between EF and academic achievement. The association between EF and achievement was negative, but simple slopes analysis revealed that this was only significant at a low level of ADHD traits (*p* < 0.006). At average and high levels of ADHD traits, the interaction was non-significant. At a high level of ADHD traits, the relationship between EF and achievement was positive, but as this was non-significant it cannot be reliably interpreted.

With DCD traits as the moderator, 5% of the variance in academic achievement was explained, *R*^2^ = 0.05, *F*(3, 154) = 2.87, *p* = 0.038. The interaction between executive function and DCD traits was significant [β = 0.02, *t*(154) = 2.38, *p* = 0.019], suggesting that DCD traits significantly moderated the relationship between EF and academic achievement. The association between EF and achievement was negative, but simple slopes analysis revealed that this was only significant at a low level of DCD traits (*p* < 0.005). At average and high levels of DCD traits, the interaction was non-significant. At a high level of DCD traits, the relationship between EF and achievement was positive, but as this was non-significant it cannot be reliably interpreted.

## Discussion

The first aim of the current study was to assess whether traits of ASD, ADHD, and DCD could significantly predict academic achievement in university students, with the hypothesis that higher levels of traits would significantly predict lower academic achievement. ASD, ADHD, and DCD traits did not significantly predict achievement, suggesting that having a high level of traits of these conditions does not significantly predict that a student will have lower achievement. Thus, the hypothesis was not supported. Although the direction of this relationship was in line with the findings of previous studies such as St John et al. (2018), Milgramm et al. (2021), and Smits-Engelsman et al. (2021), this cannot be reliably interpreted as the model was non-significant.

Furthermore, the relationship between these variables was weak, which suggests that having a high level of autistic traits may have less of an influence on academic achievement than having a clinical diagnosis of ASD and/or DCD does. Further to this, the relationship between ADHD traits and achievement was not only non-significant, but also positive. This was the opposite direction to what was hypothesized based on previous literature such as Carrasco et al. (2021), which further suggests that the model is a poor fit.

It should be noted, however, that the current study did not specifically recruit participants with a clinical diagnosis of ASD, ADHD, or DCD; rather, the majority were neurotypical university students, although 21 of the 158 participants did have a diagnosis of at least one neurodevelopmental condition. As the study was looking at traits in a majority neurotypical sample, this may be the reason that levels of ASD, ADHD, and DCD traits did not significantly predict academic achievement as hypothesized.

Additionally, many of the studies that have identified poorer academic performance in individuals with neurodevelopmental disorders studied children, such as Kim et al. (2018); Fastame (2020), Milgramm et al. (2021), and Smits-Engelsman et al. (2021), whereas the present study was conducted with adult university students. This may explain why ASD, ADHD, and DCD traits did not significantly predict academic achievement, as it is possible that individuals with these conditions may experience a reduction in the severity and impact of their condition on many areas of their life with age (Adler and Chua, 2002; Shattuck et al., 2007; Esbensen et al., 2009).

The non-significant relationship between ASD, ADHD, and DCD traits and achievement could also perhaps be explained by the possibility that some of the students who participated in this study may have received additional academic support, which could positively affect their academic performance. In addition, those with higher levels of traits but not high enough for a clinical diagnosis may not experience significant academic difficulty. This may be why these individuals do not reach the threshold for a clinical diagnosis; the level of traits they report may not have enough of a significant impact on their daily life that their academic performance is affected, which can be the reason why many individuals with ADHD, for example, are brought to the attention of clinicians to begin with (Loe and Feldman, 2007). However, it is also possible that ASD, ADHD, and DCD traits alone did not significantly predict achievement as there is another factor influencing the relationship—executive function—as the moderation analysis revealed.

The second aim was to determine whether traits of ASD, ADHD, and DCD moderate the relationship between executive function and academic achievement, with the hypothesis that higher levels of ASD, ADHD, and DCD traits would significantly moderate a positive relationship between executive function and academic achievement. ASD traits did not significantly moderate the relationship between EF and academic achievement, but ADHD and DCD traits did significantly moderate this relationship. However, the interaction was only significant at lower levels of ADHD and DCD traits, and the relationship between executive function and achievement was negative. This suggests that lower levels of ADHD and DCD traits, respectively, significantly moderated a negative relationship between executive function and achievement therefore the second hypothesis is not fully supported despite the statistical significance.

These findings do not provide adequate support for the findings of Gropper and Tannock (2009), who found that working memory impairments can negatively affect academic achievement in university students with ADHD. However, it should be noted that Gropper and Tannock (2009) did not investigate the moderating role of the levels of ADHD traits, so the findings of the present study are not directly comparable. Further to this, in both the moderation analyses with ADHD and DCD traits as the moderators, respectively, the relationship between executive function and achievement was negative, which does not align with the findings of numerous previous studies. The majority of studies that found a positive relationship between executive function and academic achievement have been conducted on children, so the results of the current study could suggest that executive function may not be as strong a predictor of academic achievement in adult students than it is in children.

It is also worth noting that the relationship between executive function and achievement was only negative at lower and average levels of both ADHD and DCD traits. At higher levels of ADHD and DCD traits, this relationship was positive, which, although statistically non-significant, supports the direction of the second hypothesis. Despite the statistical non-significance, this does not suggest that there is no relationship between these variables. Rather, this may suggest that for some neurotypical individuals, having a high level of ADHD and/or DCD traits and poorer executive function could potentially be a risk factor for lower academic performance. However, additional research would be needed to explore this possibility further, and investigate whether or not this result is repeated and significant in a larger number of students from a wider variety of higher education institutions. Such findings may be of prognostic value; academic support could be put in place earlier for students with poorer executive functioning and higher levels of ADHD and/or DCD traits to promote academic success. It should also be noted that the percentage of variance in academic achievement accounted for by EF and traits of ASD, ADHD, and DCD, respectively, is fairly low, which could suggest that there is another factor that could account for variance in achievement that was not measured in the present study.

The current study can offer a contribution to the gap in the literature concerning the role of neurodevelopmental disorder traits in the relationship between executive function and achievement in the adult student population, and the nature of the relationship between these variables in neurotypical students. This study also contributes evidence of a moderating role of ADHD and DCD traits in the relationship between EF and achievement in neurotypical university students, suggesting that the relationship between EF and neurodevelopmental disorder traits in relation to academic performance may not only be limited to individuals with a clinical diagnosis of a neurodevelopmental condition. However, further research is needed to support this suggestion, as research into this understudied area is limited and therefore reduces the extent to which the findings can be reliably interpreted and compared with findings of previous research.

The current study also offers evidence to suggest that students with a high level of neurodevelopmental disorder traits but no clinical diagnosis may not potentially be as at risk of academic underachievement compared with individuals who have a clinical diagnosis of ASD, ADHD, or DCD. However, this does not necessarily mean that no neurotypical university students with a high level of ASD, ADHD, or DCD traits will ever be at risk of academic underachievement. ASD and DCD traits displayed a negative relationship with academic achievement, and, although non-significant, there could be a possibility that individual students with a high level of ASD or DCD traits, who do not reach the threshold for a clinical diagnosis, could face academic difficulty. With no clinical diagnosis, they may not come to the attention of university academic support services, potentially placing them at risk of academic underachievement.

Despite the contributions of this study, it has some noteworthy limitations. Firstly, executive function and DCD traits were measured using a self-report questionnaire rather than lab-based assessments. The Amsterdam Executive Function Inventory (AEFI; Van der Elst et al., 2012) and the Adult DCD Checklist (Kirby et al., 2010) were used in the present study for the convenience of being able to administer the measures online to a large number of university students, and for the relevance of its items to everyday life. However, using an executive function battery comprised of various widely used tasks, such as verbal fluency, the Wisconsin card sorting test, and the digit span test (Faria et al., 2015) for example, may be a better measure for future research into this area.

More recently, the Free Research Executive Function Evaluation (FREE; Zanini et al., 2021) test battery for executive function has been developed; which is designed to be completed at the individual’s own pace, so that performance is not affected by speed of perception or psychomotor ability. Zanini et al. (2021) also conclude that the FREE battery can be administered to populations other than and including Western cultures, and to individuals of any socioeconomic status, suggesting that the FREE may have huge potential as a global measure of executive function. In addition to lab-based methods, future research should also include a self-report measure such as the AEFI (Van der Elst et al., 2012), or the Behavior Rating Inventory of Executive Function (BRIEF; Gioia et al., 2000), to provide data on executive function using items that relate more closely to everyday activities than the lab-based tasks, which offers higher ecological validity (Leonard and Hill, 2015).

For the DCD checklist, the first section contains items regarding childhood, which gathers retrospective data. This could mean that these answers may no longer be relevant to the participants’ current motor skills, potentially affecting the accuracy of the results. To address this limitation, a lab-based task could be used to measure motor skills in addition to the self-report measure of DCD traits, such as the Test of Motor Competence (TMC), an assessment battery that assesses both gross and fine motor skills, as described by Sigmundsson et al. (2016). Using a direct measure of motor co-ordination specifically rather than simply measuring DCD traits could provide an insight into the potential relationship between executive function, motor co-ordination, and academic performance, for which there is some similar evidence from Fastame (2020), who concluded that motor co-ordination is crucial for academic achievement in both neurotypical individuals and those with a clinical diagnosis of DCD.

Another limitation of the present study was that the majority of participants were female. Research has suggested that a higher number of males have ASD compared to females (Loomes et al., 2017; Ratto et al., 2018), and that females with ASD may be more likely to “mask” their autistic behaviors (Ratto et al., 2018; Pearson and Rose, 2021). Therefore, a sample of mostly female participants could mean that any potential relationships between ASD traits and academic achievement may appear non-significant. It is also considered that there is a great deal of heterogeneity in autism (Lenroot and Yeung, 2013; Jeste and Geschwind, 2014; Pearson and Rose, 2021), therefore, it may be possible that not all individuals with a high level of autistic traits experience the same traits to the same degree. This variation may lead to non-significant findings in the relationship between ASD traits and academic achievement, and may result in any potential moderating role of ASD traits appearing non-significant in the relationship between executive function and academic achievement.

A further notable limitation is that the study did not require participants to disclose which institution they were studying at, or which course they were on. The majority of the participants were studying at the researcher’s affiliated institution, which means that these results mainly reflect the responses of university students studying in the United Kingdom in one institution. The study was also available to students studying at universities in other countries, therefore, the study has not taken into account any potential influences of cultural factors or socioeconomic status. It may have also been useful to know which courses the participants were studying, in order to account for the possibility that some students may not be studying “academic” subjects, and may not be graded using the percentage format.

The current study also did not consider whether participants had accessed any assistance from university academic support services or if they were in receipt of additional examination requirements such as extra time. Accessing such support may positively affect students’ academic performance which may render any relationship between neurodevelopmental disorder traits and academic achievement non-significant. To address this limitation, future research should note whether participants have accessed academic skills support and whether they receive any additional examination requirements, and if so, which. This could also provide data on the role of academic support as a possible protective factor in the relationship between neurodevelopmental disorder traits and academic achievement. Future research could explore whether such interventions can improve academic achievement outcomes in university students with a high level of neurodevelopmental disorder traits, as well as those with a clinical diagnosis.

As it is consistent among the literature that executive function can be impaired and academic achievement can be lower in individuals with ASD, there may be a possibility that while the interaction between executive function and ASD traits was non-significant in a sample of majority neurotypical students, this relationship may be significant in individuals with a clinical diagnosis of ASD. This may be especially relevant as the result was very close to the significance threshold, thus, future research with a clinical sample may find a significant result. Therefore, future research could explore the potential moderating role of ASD traits on the relationship between executive function and achievement in university students with a clinical diagnosis of ASD, and perhaps other conditions known to affect academic performance in university students, such as dyslexia (Richardson, 2010; Stagg et al., 2018).

It may also be worth investigating the possibility of a different relationship between executive function, neurodevelopmental disorder traits and academic achievement; for example, the potential mediating role of executive function in the relationship between neurodevelopmental disorder traits and academic achievement in university students. There is evidence for this potential relationship coming from similar studies by Cadoret et al. (2018) with children with DCD, and Schmidt et al. (2017) with typically developing children, who found that executive function had a significant mediating role in the relationship between children’s motor co-ordination and academic achievement.

A further possible direction for future research could be to consider the roles of traits that overlap between neurodevelopmental disorders, and of co-occurrence of such conditions, in academic achievement. It is possible for neurodevelopmental disorders such as ASD, ADHD, and DCD to co-occur (Tal-Saban and Kirby, 2019; Miller et al., 2021), and there are often traits that are found in more than one of these conditions (Reiersen et al., 2008), for example, motor difficulties can be found in ASD and ADHD as well as DCD (Lindor et al., 2019; Miller et al., 2021). The current study examined whether traits of ASD, ADHD, and DCD could separately moderate the relationship between executive function and academic achievement, so future research could assess whether traits of these disorders together can moderate this relationship in students with clinical diagnoses, which could have notable implications for individuals who have been diagnosed with co-occurring ASD, ADHD, or DCD. There appears to be scope for this suggestion, given the increasing popularity of the transdiagnostic approach to neurodevelopmental disorders, as discussed by Astle et al. (2022). Such research could highlight the need for further research into effective academic support interventions for these students, to enable them to perform at the best of their capabilities.

## Conclusion

The results of this study appear to suggest that neurodevelopmental disorder traits alone do not significantly predict achievement. This may provide evidence that also relates to clinical samples; the poorer academic achievement associated with neurodevelopmental disorders may be influenced by factors other than the traits of the neurodevelopmental conditions themselves. However, when ADHD and DCD traits, respectively, interact with executive functioning, they do significantly predict achievement. Thus, the relationship between neurodevelopmental disorder traits and academic achievement may only be significant in neurotypical individuals when executive function is taken into account.

However, it may still be possible for individual students with a high level of traits to be at risk of academic underachievement—students with a high level of ADHD or DCD traits and poor executive function may be at risk of lower achievement, although further research is needed to determine whether this result would be replicated and significant in a larger sample of students from a wider range of higher education institutions. Furthermore, as executive function displayed a negative relationship with achievement, this could suggest that executive function may be more of a significant contributor to academic achievement in children than in adult students, however, this area still remains understudied in adults. Given that the percentage of variance in academic achievement accounted for by executive function and traits of ASD, ADHD, and DCD, respectively, was low, this may suggest that there is a possibility of another factor influencing achievement. Future studies that seek to explore this topic further should incorporate laboratory-based methods when assessing executive function in addition to self-report measures relating to everyday tasks, to provide a thorough and reliable insight into the nature of the relationship between executive function, neurodevelopmental disorder traits, and academic achievement.

## Data availability statement

The raw data supporting the conclusions of this article will be made available by the authors, without undue reservation.

## Ethics statement

The studies involving human participants were reviewed and approved by the Psychology and Counselling Departmental Ethics Committee, University of Greenwich, London, United Kingdom. The patients/participants provided their written informed consent to participate in this study.

## Author contributions

CS contributed to the planning, design, data collection and analysis, and write-up of this research.
